# NGF receptors and PI3K/AKT pathway involved in glucose fluctuation-induced damage to neurons and α-lipoic acid treatment

**DOI:** 10.1186/s12868-020-00588-y

**Published:** 2020-09-17

**Authors:** Ting Yan, Zhihui Zhang, Danqing Li

**Affiliations:** 1 Department of Endocrinology, Huai’an Cancer Hospital, Huaian, Jiangsu China; 2 Shanghai TCM-Integrated Hospital, Shanghai University of Traditional Chinese Medicine, Shanghai, China; 3 Shanghai TCM-Integrated Institute of Vascular Anomalies, Shanghai, China; 4 Department of Endocrinology, The Second Hospital Affiliated To Dalian Medical University, Dalian, China

**Keywords:** Diabetic encephalopathy, TrkA, p75NTR, PI3K/AKT, Intermittent high glucose, Cell apoptosis

## Abstract

**Background:**

Glucose fluctuation promotes neuronal apoptosis, which plays a central role in diabetic encephalopathy (DE). Nerve growth factor (NGF), and its interaction with high-affinity (TrkA) and low-affinity (p75NTR) receptors, are involved in neuronal survival. NGF/TrkA contributes to the activation of the PI3K/AKT pathway, which is beneficial for neuronal survival, and α-Lipoic acid (ALA) exerts clinically favorable neuroprotective effects in the periphery. Whether NGF receptors and the PI3K/AKT pathway are involved in glucose fluctuation-induced neuronal damage, as well as the potential molecular mechanism of ALA in protecting glucose fluctuation-induced neuronal damage, remain unclear.

**Results:**

The results indicated that constant high glucose (CHG) and intermittent high glucose (IHG) significantly increased the expression of Bax and caspase-3, and decreased the expression of TrkA/p75NTR and p-AKT/AKT, while ALA stimulation reversed the above proteins in PC12 cells. IHG stimulates apoptosis more effectively than CHG in PC12 cells, which is related to the PI3K/AKT pathway but not to the TrkA/p75NTR. Furthermore, neuronal apoptosis induced by IHG was aggravated by the TrkA inhibitor K252a or the PI3K/AKT inhibitor LY294002, but this effect was alleviated by the p75NTR inhibitor TAT-pep5.

**Conclusion:**

Glucose fluctuation induced cell apoptosis by regulating the TrkA/p75NTR and PI3K/AKT pathway, meanwhile ALA exhibited neuroprotective effects in response to IHG and CHG. These observations indicated that the PI3K/AKT pathway and the balance of TrkA/p75NTR are likely to serve as potential therapeutic targets for DE. In addition, ALA could be a possible therapeutic drug for DE.

## Background

DE is a chronic complication of type 2 diabetes mellitus (T2DM) that is characterized by cognitive impairment and dementia [23]. With the increasing lifespan of patients with diabetes, the risk of cognitive impairment and dementia is also increasing [36]. In the management of diabetes, poor diet control, improper treatment of insulin and hypoglycemic drugs, and inadequate patient compliance can lead to increased glucose variability [33]. Therefore, glucose fluctuation, which is independent of the average blood glucose level [22], is ubiquitous in patients with diabetes, and promotes the development of DE [11, 31].

Although many clinically available drugs are effective at controlling blood glucose level, glucose fluctuation is difficult to avoid. Therefore, it is particularly important to establish an effective approach to alleviate cognitive impairment in patients with diabetes under glucose fluctuation. Some studies have demonstrated that neuronal apoptosis plays a vital role in the development of cognitive dysfunction [4, 13]. However, the molecular mechanism underlying glucose fluctuation promoting cell apoptosis is unclear.

NGF plays an important role in the plasticity and survival of basal forebrain cholinergic neurons associated with cognitive function. It exerts its physiological effects by binding two classes of cell surface receptors, the high-affinity tropomyosin-related kinase A (TrkA) receptor and the low-affinity p75 neurotrophin receptor (p75NTR), between which there are complex interactions. NGF/TrkA mainly promotes pro-survival signals to protect cholinergic neurons. In contrast, stimulation of the p75NTR receptor activates cell apoptosis, but increases the affinity and selectivity of NGF binding to TrkA in the presence of TrkA [3]; the p75NTR/NGF/TrkA signaling pathway promotes neuronal survival [45]. In addition, p75NTR can delay the internalization and degradation of TrkA following NGF treatment to promote the elongation of the TrkA signal on the cell surface [28]. Therefore, disorders of NGF and its receptors are key factors in cholinergic neuronal dysfunction in patients with cognitive impairment [20]. A previous study indicated that TrkA reduction occurred from non-cognitive impairment to mild cognitive impairment, and from mild cognitive impairment to veritable Alzheimer’s disease (AD) in humans [16]. Furthermore, TrkA reduction in the cerebral cortex is positively correlated with cognitive decline based on the Mini-Mental State Examination (MMSE) test scores [10]. The average level of p75NTR in the hippocampus of patients with dementia was significantly higher than that of patients without dementia of the same age [7, 47]. Moreover, cognitive function and spatial memory were improved in p75NTR knockout mice [1]. Hence, the imbalance of NGF receptors may be one of the risk factors for cognitive impairment, and targeting NGF receptors may delay cognitive disorders [32]. The NGF/TrkA complex activates downstream signaling pathways, such as phosphatidylinositol 3 kinase/protein kinase B (PI3K/AKT), which is involved in the pathological process of cognitive dysfunction [43]. In addition, the PI3K/AKT pathway plays a pivotal role in the inhibition of diabetes-induced neuronal apoptosis [12, 42], and participates in the regulation of retrograde axonal transport of neurotrophins in the nervous system [5]. In contrast, dysfunction of the PI3K/AKT pathway can induce metabolism of the Aβ peptide, resulting in the phosphorylation and accumulation of Tau protein, which leads to the occurrence and development of cognitive dysfunction [32].

ALA is a widely available dietary supplement [48] and a clinical drug for the treatment of diabetic peripheral neuropathy. ALA, and its reduced form, dihydrolipoic acid (DHLA), exert neuroprotective effects through various pathways, including increased glucose uptake [18] and acetylcholine production [19], and modulation of signaling pathways for nuclear factor kappa B (NF-κB) and insulin [17]. However, it remains unknown whether ALA can exert its neuroprotective effects under glucose fluctuation. This study aimed to investigate the underlying molecular mechanism of neuronal damage caused by glucose fluctuation in vitro, and whether ALA protects neuronal cells under glucose fluctuation.

## Results

### Determination of high glucose and ALA concentrations

**Fig. 1:**
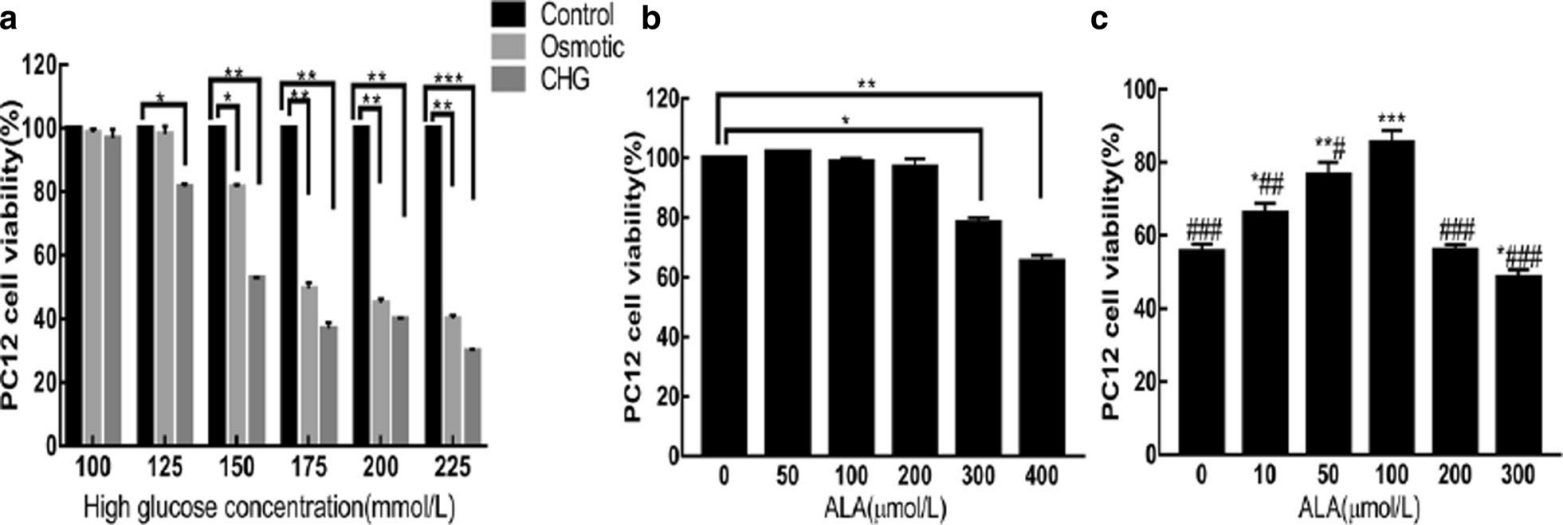
**a** Effect of glucose on PC12 cell viability. **b** Effects of ALA on PC12 cell viability under normal glucose concentration. **c** Effects of ALA on PC12 cell viability under glucose fluctuation. Results are presented as the mean ± SD (n = 3). ***P < 0.001, **P < 0.01,*P < 0.05, compared to the (**a**) control or (**b**, **c**) ALA (0 µmol/L) group. ^###^P < 0.001, ^##^P < 0.01, ^#^P < 0.05 versus the ALA (100 µmol/L) group

To obtain the high glucose concentration in the fluctuation range, PC12 cells were cultured in increasing concentrations of glucose (from 100–225 mM) for 72 h. Equal concentration of mannitol was used as a negative control for osmolarity. CHG inhibited PC12 cell viability in a concentration-dependent manner. As shown in Fig. 1a, mannitol could influence cell viability when the osmolarity increased to 150 mM. Thus, we chose 125 mM as the concentration of high glucose in glycemic fluctuation. Subsequently, PC12 cells were cultured in 25 mM glucose medium containing different doses of ALA (0–400 µmol/L) for 48 h. We found that 300 and 400 µmol/L ALA showed toxic effects in PC12 cells (Fig. 1b). Finally,PC12 cells were exposed to glucose alternation between 25 mM and 125 mM for 72 h after pretreatment with ALA (0–300 µmol/L) for 24 h. We found that 100 µmol/L ALA was the optimum concentration, which induced a more evident effect on PC12 cell viability, and was used in the following experiments (Fig. 1c).

### ALA treatment attenuated IHG-induced cell injury in PC12 cells

**Fig. 2:**
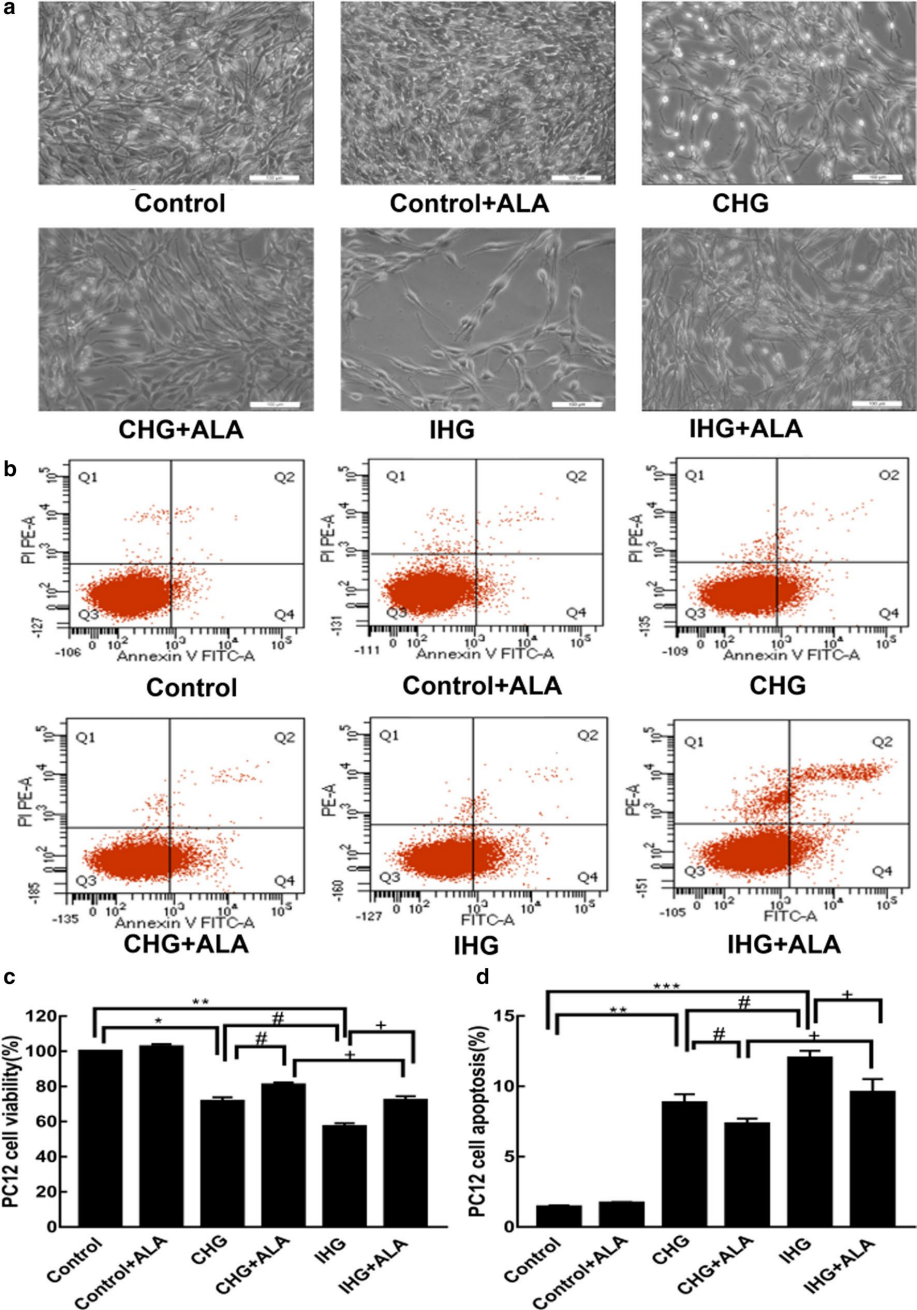
ALA treatment attenuated IHG induced cell injuries in PC12 cells. **a**, **c** Effects of IHG on PC12 cell viability and ALA treatment. Cells were observed by microscope, and viability was determined by MTT assay. **b**, **d** Effects of IHG on PC12 cell apoptosis and ALA treatment. Apoptosis was detected by flow cytometry. Results are shown as the mean ± SD (n = 3). ***P < 0.001, **P < 0.01,*P < 0.05 versus the control; ^#^P < 0.05 compared to the CHG group; and ^+^P < 0.05 versus the IHG + ALA group

IHG-induced cell damage is more serious than that induced by CHG. However, ALA significantly alleviated the IHG-induced decrease in the cell amount and viability, which was weaker than the effect of ALA on CHG-induced cell damage (Fig. 2a, c). We further explored IHG-induced cell apoptosis by flow cytometry, and found that IHG-induced apoptosis was more obvious than that observed with CHG. Moreover, ALA significantly repressed IHG- and CHG-induced apoptosis, as evidenced by the decreased apoptotic rate (Fig. 2b, d).

### ALA treatment inhibited IHG-induced apoptosis in PC12 cells

**Fig. 3:**
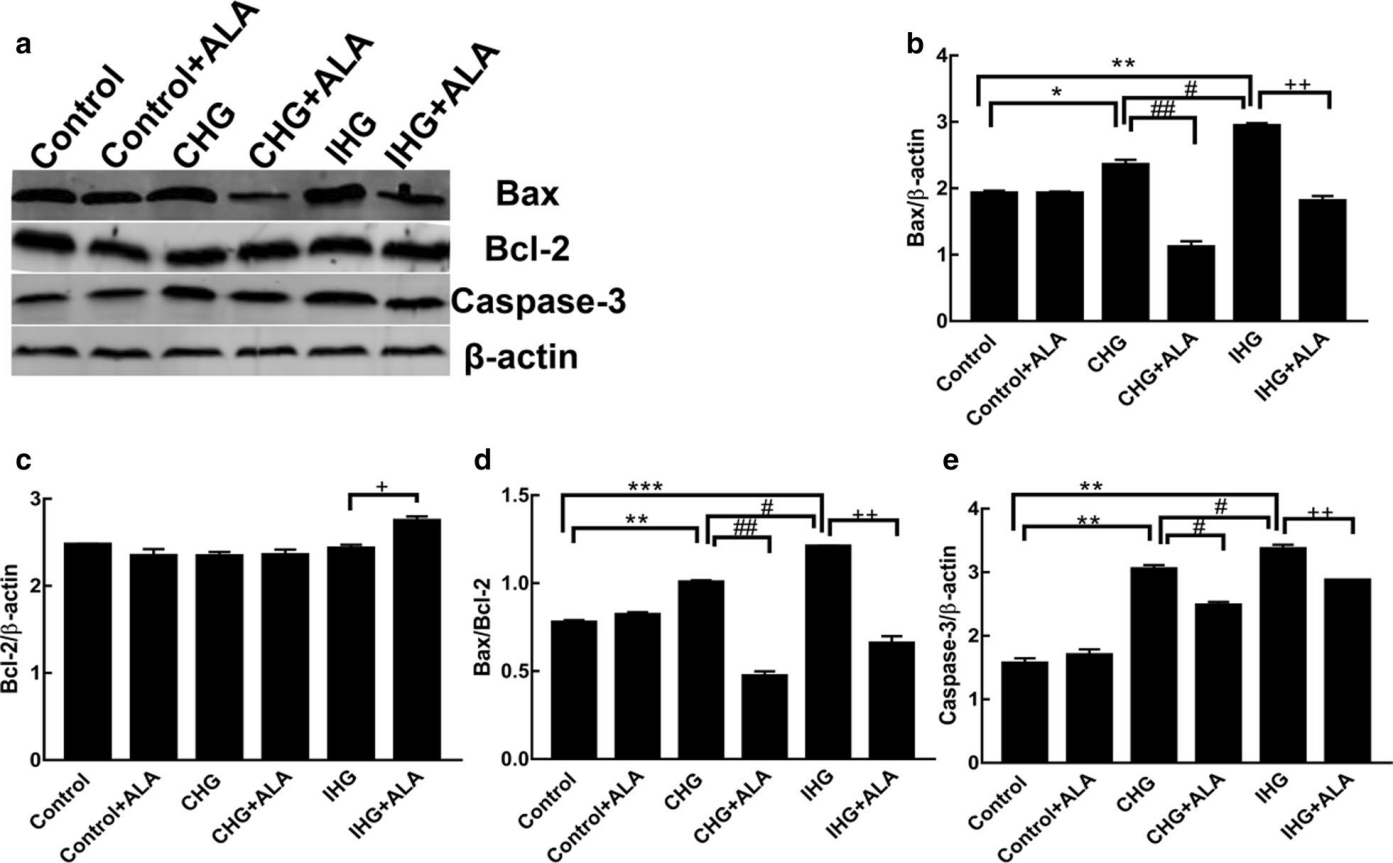
ALA treatment inhibited IHG-induced apoptosis in PC12 cells. The density of (**a**) Bax, Bcl-2, and Caspase-3 were measured by Western blot. Quantitative data of (**b**) Bax, (**c**) Bcl-2, (**d**) Bax/Bcl-2, and (**e**) Caspase-3 are presented. Data are shown as mean ± SD (n = 3). ***P < 0.05, **P < 0.01, *P < 0.05 versus the control; ##P < 0.01, #P < 0.05 compared to the CHG group; and ^++^P < 0.01, ^+^P < 0.05 versus the IHG + ALA group

With regard to the pro-apoptotic effect of IHG, we found that both CHG and IHG elevated Bax and Caspase-3, but that IHG was more effective than CHG in promoting pro-apoptotic protein expression. Neither had an effect on Bcl-2; however, IHG-induced apoptosis was significantly attenuated by ALA treatment, as evidenced by decreased Bax, Caspase-3, and increased Bcl-2 (Fig. 3). This indicated that ALA promoted the survival of neuronal cells exposed to IHG, which was not significantly inferior to the effect of ALA on CHG.

### Effects of ALA treatment on IHG-induced NGF receptors in PC12 cells

**Fig. 4:**
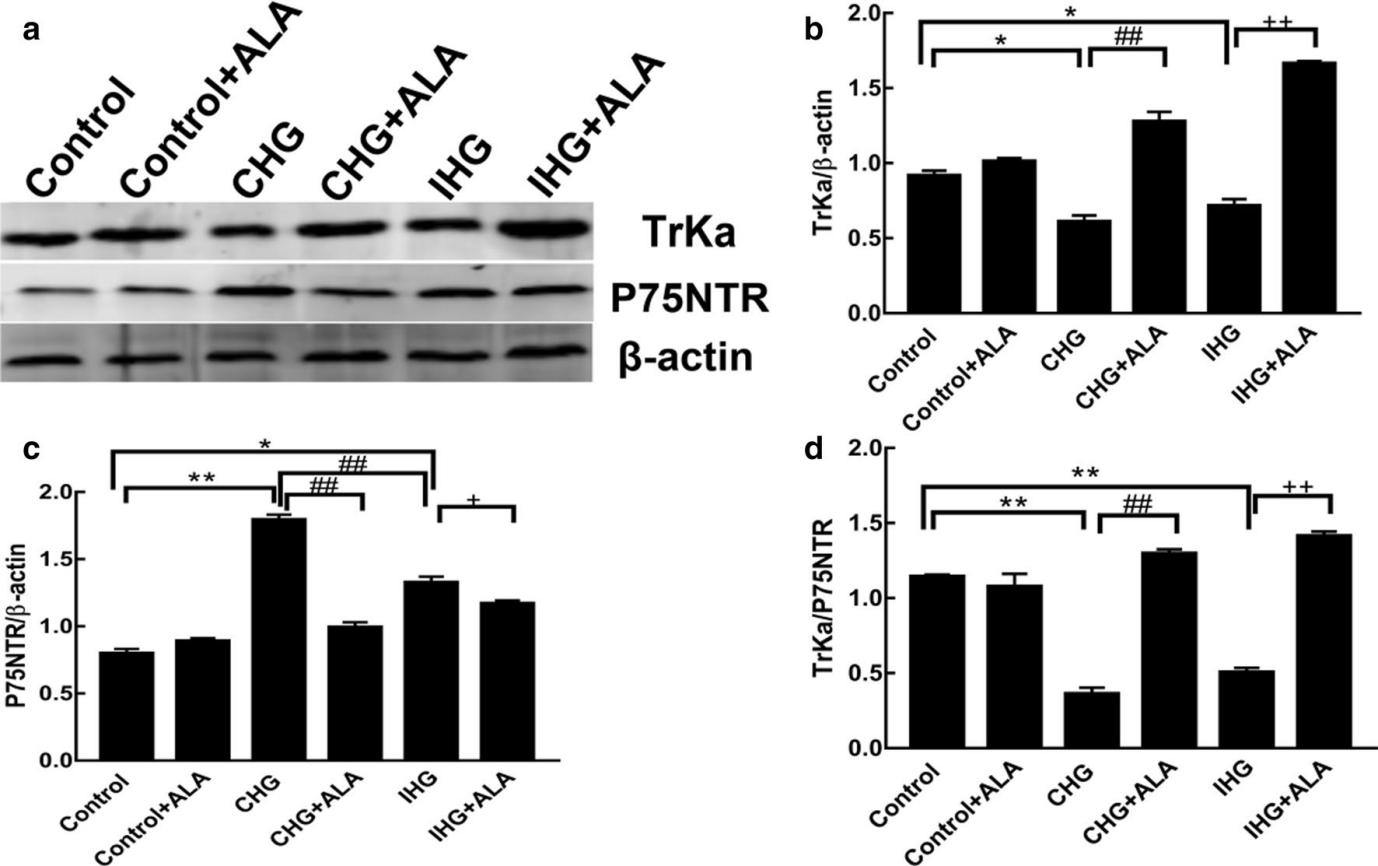
ALA treatment affected IHG-induced NGF receptorsin PC12 cells. **a** The density of TrkA and p75NTR were determined by Western blot. Quantitative data of (**b**) TrkA, **c** p75NTR, and **d** TrkA/p75NTR are presented. Data are presented as mean ± SD (n = 3). **P < 0.01,*P < 0.05 compared to the control; ^##^P < 0.01 versus the CHG group; and ^++^P < 0.01, ^+^P < 0.05 compared to the IHG + ALA group

Further studies showed that IHG and CHG significantly decreased TrkA and TrkA/p75NTR, and increased p75NTR, but had no significant difference on TrkA/p75NTR. Interestingly, ALA pretreatment reversed the effects of IHG and CHG on TrkA and p75NTR in PC12 cells (Fig. 4).

### ALA treatment altered the IHG-inhibited PI3K/AKT pathway in PC12 cells

**Fig. 5:**
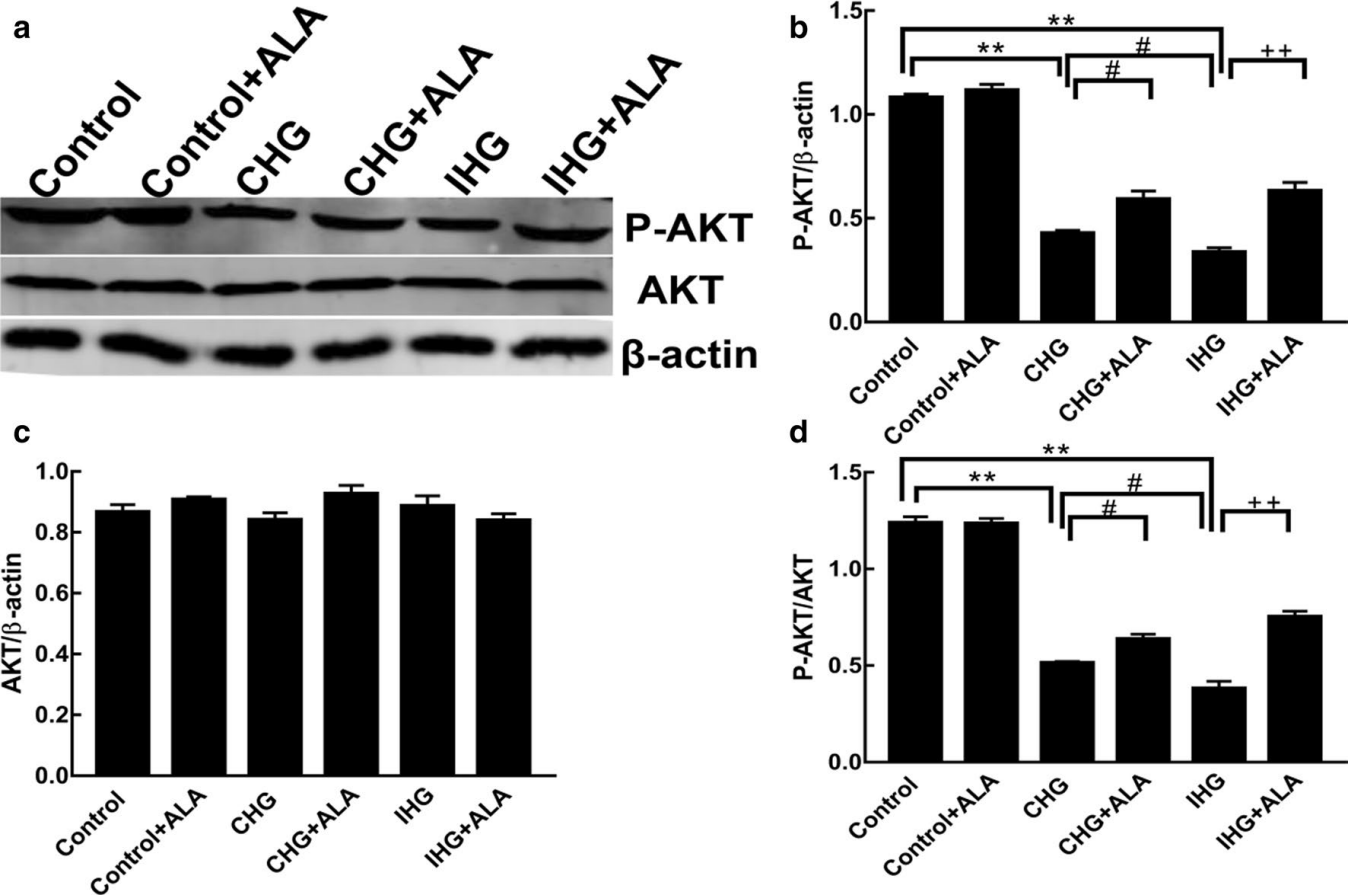
ALA treatment altered the IHG-inhibited PI3K/AKT pathway in PC12 cells. **a** The density of AKT and p-AKT were measured by Western blot. Quantitative data of **b** p-AKT, **c** AKT, and **d** p-AKT/AKT are presented. Data are shown as mean ± SD (n = 3). **P < 0.01 compared to the control; ^#^P < 0.05 compared to the CHG group; and ^++^P < 0.01 versus the IHG + ALA group

We measured the levels of p-AKT and AKT to explore whether IHG-mediated cell apoptosis was related to the PI3K/AKT pathway. Both CHG and IHG reduced p-AKT and p-AKT/AKT, but had no significant effect on AKT. Meanwhile, the effect of IHG on lowering p-AKT/AKT was greater than that of CHG. However, the inhibitory effects of IHG on p-AKT and p-AKT/AKT were attenuated by ALA, which was superior to the effect of ALA on p-AKT and p-AKT/AKT induced by CHG (Fig. 5).

### NGF receptors and the PI3K/AKT pathway involved in IHG-induced cell apoptosis and ALA protected this process

**Fig. 6:**
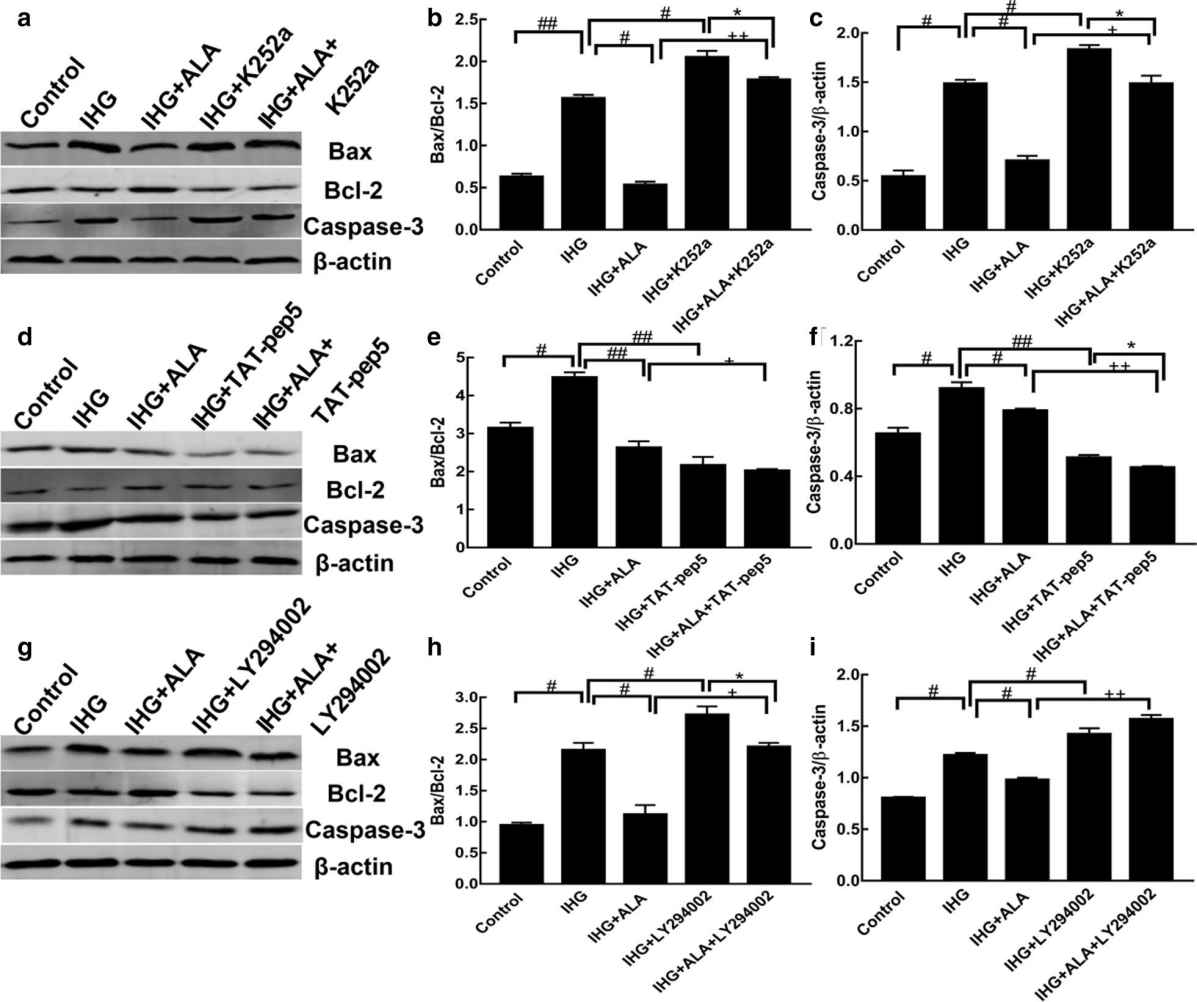
ALA protected IHG-induced cell apoptosis through NGF receptors and the PI3K/AKT pathway in PC12 cells. Bax/Bcl-2 and Caspase-3 levels were evaluated in the presence or absence of K252a (**a–c**), TAT-pep5 (**d-f**), and LY294002 (**g–i**), respectively. Data are shown as the mean ± SD (n = 3). ^##^P < 0.01, ^#^P < 0.05 versus the IHG group; ^++^P < 0.01, ^+^P < 0.05 compared to the IHG + ALA group; and *P < 0.05 compared to the IHG + inhibitor (K252a, TAT-pep5, or LY294002)

We measured apoptosis proteins in the presence or absence of K252a, TAT-pep5, and LY294002 (inhibitor of TrkA, p75NTR, and PI3K, respectively) to explore the molecular mechanism of IHG-induced cell apoptosis. As depicted in Fig. 6, pretreatment with K252a and LY294002 further aggravated IHG-induced Bax/Bcl-2 and Caspase-3 expression. Meanwhile, K252a and LY294002 significantly reversed the IHG-induced effects of ALA on Bax/Bcl-2 and Caspase-3. Meanwhile, TAT-pep5 reduced the levels of Caspase-3 and Bax/Bcl-2 induced by IHG. Moreover, TAT-pep5 further enhanced the effect of ALA on reducing Bax/Bcl-2 and Caspase-3 in IHG conditions.

## Discussion

Poor diet control, improper treatment with insulin and hypoglycemic drugs, and inadequate compliance are associated with blood glucose fluctuation during the course of T2DM [33]; thus, glucose fluctuations are difficult to avoid in patients with diabetes. Several studies have demonstrated that IHG-induced damage is more serious than CHG in vascular endothelial cells, olfactory neuroepithelial cells, retinal epithelial cells, and Schwann cells [15, 21, 38, 44]. However, the effects of IHG on neuronal cells and their potential mechanisms are unknown.

Both high glucose-mediated metabolic stress in the mitochondria and extracellular high glucose-mediated osmotic stress result in the activation of apoptotic pathways [6, 29, 34, 39]. In order to elucidate which of these mechanisms was most related to the observed effects, mannitol and glucose at the same concentration were used to reproduce the effects of osmolarity on neurons. A glucose concentration with high glucose-mediated metabolic stress but no osmolarity was used as a high value of glucose fluctuation.

In our study, both CHG and IHG led to decreased cell viability and increased cell apoptosis; this effect was more obvious in the IHG group. This was similar to previous studies, which indicated that glucose fluctuation was more harmful than high glucose in promoting diabetes complications [24]. Bax and Bcl-2 are key regulators of the mitochondrial apoptotic pathway [8]. Bcl-2 can prevent cytochrome C release from the mitochondria to the cytoplasm to inhibit apoptosis, while Bax promotes apoptosis and antagonizes the protective effect of Bcl-2. In addition, Caspase-3 is a cysteine protease that is involved in the regulation of neuronal apoptosis [35]. Our research showed that both IHG and CHG induced neuronal damage and cell apoptosis by increasing Bax and Caspase-3, although the effect was more obvious in IHG.

The maturation, plasticity, and survival of cholinergic neurons are inseparable from the nutritional support of NGF. NGF exerts physiological effects by binding to the TrkA or p75NTR receptor, while TrkA signaling inhibits apoptosis and promotes neuronal survival [49]; in contrast, the intracellular domain of p75NTR contains a “death domain,” and p75NTR overexpression facilitates neuronal apoptosis [2, 14]. Therefore, we explored the effects of IHG and CHG on NGF receptors due to the close relationship between NGF receptors and neuronal apoptosis, and found that both IHG and CHG decreased TrkA level. Studies have shown that IHG reduces the release of neuronal insulin-like growth factor 1 (IGF-1) [15], while IGF-1 could increase TrkA [25] level; therefore, IHG might reduce TrkA expression through the IGF-1 pathway [9]. In the current study, IHG and CHG increased p75NTR in PC12 cells. This result is similar to that observed in a clinical study, which found that the p75NTR level is twice as high in patients with cognitive disorders when compared with in age-matched individuals with no cognitive disorders [7]. To further investigate the role of NGF receptors in IHG-induced neuronal apoptosis, we found that K252a (a TrkA inhibitor) further aggravated the effect of IHG on apoptosis protein, and that TAT-pep5 (a p75NTR inhibitor) reversed the Caspase-3 expression induced by IHG, but had no significant effect on Bax/Bcl-2. These findings indicate that IHG promoted neuronal apoptosis through TrkA and p75NTR receptors. Previous studies have reported that p75NTR increases the affinity and selectivity of NGF binding to TrkA in the presence of TrkA [3], and that the p75NTR/NGF/TrkA signaling pathway promotes neuronal survival [45]. TrkA deletion may cause an increase in the binding of pro-nerve growth factor (proNGF) to p75NTR, which transforms neuronal survival into pro-apoptotic signaling [7]. In addition, p75NTR could delay the internalization and degradation of TrkA following NGF treatment to promote the elongation of TrkA signaling [28]. TrkA/p75NTR served as an evaluation index to more clearly represent the trend of NGF receptors under IHG. In this study, although both IHG and CHG reduced TrkA/p75NTR, they did not differ significantly, suggesting that the difference in neuronal apoptosis caused by IHG and CHG might not be related to TrkA/p75NTR level.

Research has shown that NGF/TrkA signaling induces activation of the PI3K/AKT pathway. AKT, a key molecule regulating the survival of neurons [46], reportedly facilitates cell survival via a phosphorylation cascade that blocks the insertion of pro-apoptotic Bax into the outer mitochondrial membrane, and prevents the destruction of mitochondrial membrane potential, the release of cytochrome C, and apoptosis signaling [37]. Our results showed that both IHG and CHG decreased p-AKT and p-AKT/AKT in PC12 cells, which was similar to that CHG reduced phosphorylation of AKT observed in nerve cells [26], and that IHG decreased p-AKT/AKT in human umbilical vein endothelial cells [40]. LY294002, a PI3K inhibitor, further aggravated the effect of IHG on Bax/Bcl-2 and Caspase-3, suggesting that IHG might cause neuronal apoptosis through the PI3K/AKT pathway. In addition, IHG induced neuronal apoptosis to a greater extent than CHG. This finding suggests that the difference in neuronal apoptosis caused by IHG and CHG might be related to the PI3K/AKT pathway, which may explain the different degrees of neuronal apoptosis caused by CHG and IHG.

ALA is a potent antioxidant that exists in the human diet [48], and has been reported to be able to pass through the blood–brain barrier to exert neuroprotective effects. Therefore, ALA is considered an ideal drug for the treatment of central nervous system diseases [48]. Our results showed that ALA repressed IHG- and CHG-induced neuronal apoptosis by affecting apoptosis protein expression. In addition, K252a and LY294002 reversed the effects of ALA on Bax/Bcl-2 and Caspase-3 under IHG, but TAT-pep5 could further reduce ALA-lowering Bax/Bcl-2 and Caspase-3 under IHG. This result suggests that ALA inhibits the expressions of Bax/Bcl-2 and Caspase-3 through NGF receptors and the PI3K/AKT pathway to reduce neuronal apoptosis and promote neuronal survival.

## Conclusions

Taken together, both IHG and CHG induce neuronal apoptosis, but this pro-apoptotic effect was amplified under IHG. TrkA/p75NTR is an important factor in neuronal apoptosis. However, the differences in IHG- and CHG-induced neuronal apoptosis were not related to TrkA/p75NTR, but were instead associated with the PI3K/AKT pathway. The TrkA/p75NTR and PI3K/AKT pathway were shown to be involved in the regulation of IHG-induced neuronal apoptosis and the neuroprotective effect of ALA under IHG. Therefore, our results could provide ideas for the development of DE drugs under IHG, and also indicate that ALA is a potential drug candidate for DE.

## Methods

### Antibody report

Rabbit Bax antibody (ab32503, 1:5000), rabbit Bcl-2 antibody (ab182858, 1:5000), and rabbit Caspase-3 antibody (ab184787, 1:5000) were purchased from Abcam (Cambridge, MA, USA). Rabbit β-actin (20536-1-AP, 1:8000; Wuhan, China) was obtained from Proteintech. Rabbit TrkA antibody (2505S, 1:1000), rabbit p75NTR antibody (4201S, 1:1000), rabbit AKT antibody (4691, 1:1000), and rabbit P-AKT antibody (4060, 1:2000) were all acquired from Cell Signaling Technology (Boston, MA).

### Cell culture and treatment

PC12 cells (donated by the School of Environment and Life Sciences of Dalian University of Technology) were maintained in Dulbecco’s modified Eagle’s medium (DMEM, SH30022.01B) supplemented with 10% fetal bovine serum (FBS, GIBCO, 16000044), 5% horse serum, and 1% penicillin/streptomycin in a humidified atmosphere of 95% air and 5% CO2 at 37 °C. PC12 cells were induced by NGF (50 ng/mL, Peprotech, 1216394) to become well-differentiated PC12 cells, which have dopaminergic properties and exhibit spindle-shaped cell morphology similar to neuronal cells [27, 30]. Differentiated PC12 cells in the exponential growth phase were used in the subsequent experiments. Normal and high glucose alternation every 12 h defined the IHG model.

PC12 cells were cultured overnight in a normal glucose medium (25 mM) [41]. The PC12 cells were treated as follows: Control group (25 mM), CHG group, IHG group (25 mM and high glucose alternating every 12 h), control + ALA group (pretreatment with 100 μΜ ALA for 24 h before incubation with 25 mM glucose), CHG + ALA group (pretreatment with 100 μΜ ALA for 24 h before CHG), and IHG + ALA group (pretreatment with 100 μΜ ALA for 24 h before IHG). Cells were incubated for 72 h.

### MTT assay for cell viability

Cell viability was determined by the 3-(4,5-Dimethylthiazol-2-yl)-2,5-diphenyltetrazolium bromide (MTT) assay. PC12 cells (8 × 10^3^ cells/well) were seeded in 96-well plates and treated as described previously for 72 h. 15 μl MTT solution (5 mg/mL, Solarbio, M8180) was added to each well and incubated for 4 h at 37 °C. Subsequently, 150 μL dimethyl sulfoxide (DMSO, Sigma, D2650) was added to dissolve the formazan crystals in each well. Finally, the absorbance was measured at 490 nm using a microplate reader (Meigu Molecular, Shanghai). The absorbance values of each group were normalized to the control group for relative quantification.

### Flow cytometry assay for apoptosis

Double staining for Annexin V/FITC and PI was performed to measure the apoptosis rate of PC12 cells. PC12 cells (4 × 10^4^ cells/well) were cultured in 6-well plates and pretreated with ALA for 24 h before stimulation with CHG or IHG for 72 h. Then, PC12 cells were harvested and washed twice with cold PBS. Subsequently, cells were suspended in 400 µl binding buffer and incubated with 5 µl Annexin V/FITC and 5 µl PI for 15 min at room temperature in the dark. The fluorescence of each sample was quantitatively analyzed by flow cytometry (FACSanto TM, BD, US).

### Western blot analysis

Following the treatments described above, PC12 cells were washed twice with cold PBS and lysed with radio immunoprecipitation assay buffer (RIPA; P0013C, Beyotime Institute of Biotechnology, China). Cell lysates were centrifuged at 12,000 rpm for 10 min at 4 °C, and the supernatant was collected for further testing. The concentration of the protein samples were detected using a BCA kit (P0010S, Beyotime, China). Equal amounts of protein were separated by 10% or 12% sodium dodecyl sulfate–polyacrylamide gel electrophoresis (SDS-PAGE) and transferred to polyvinylidene fluoride (PVDF) membranes. After 30 min of blocking with the blocking solution (Beyotime Biotechnology), the membranes were incubated with primary antibodies overnight at 4 °C, followed by fluorescent secondary antibody (1:10000; Thermo Fisher, Invitrogen) for 1 h at room temperature. Then, the protein was visualized using a fluorescence imaging scanner (LI-COR, Odyssey CLx, US). β-actin was used as an internal reference, and protein expression is shown as the ratio of the band optical intensity to that of β-actin.

### Statistical analysis

All relevant data were presented as mean ± standard deviation (SD), and SPSS 22.0 was used for data analysis. Comparisons between two groups were performed using independent two-tailed Student’s t-tests, and comparisons between more than two groups were analyzed using one-way ANOVA.

## Data Availability

The datasets generated during the current study are available from the corresponding author on reasonable request.

## Abbreviations

DE: Diabetic encephalopathy
ALA: α-Lipoic acid
CHG: Constant high glucose
IHG: Intermittent high glucose
T2DM: Type 2 diabetes mellitus
TrkA: Tropomyosin-related kinase A
p75NTR: p75 neurotrophin receptor
AD: Alzheimer’s disease
MMSE: Mini-mental state examination
PI3K/AKT: Phosphatidylinositol 3 kinase/protein kinase B
DHLA: Dihydrolipoic acid
NF-κB: Nuclear factor kappa B
NGF: Nerve growth factor
DMEM: Dulbecco’s modified Eagle’s medium
FBS: Fetal bovine serum
MTT: 3-(4,5-Dimethylthiazol-2-yl)-2,5-diphenyltetrazolium bromide
DMSO: Dimethyl sulfoxide
SDS-PAGE: Sodium dodecyl sulfate–polyacrylamide gel electrophoresis
PVDF: Polyvinylidene fluoride
IGF-1: Insulin-like growth factor 1
pro-NGF: Pro-nerve growth factor
